# Human Age Prediction Based on DNA Methylation Using a Gradient Boosting Regressor

**DOI:** 10.3390/genes9090424

**Published:** 2018-08-21

**Authors:** Xingyan Li, Weidong Li, Yan Xu

**Affiliations:** 1 Department of Information and Computer Science, University of Science and Technology Beijing, Beijing 100083, China; s20170744@xs.ustb.edu.cn (X.L.); liweidong@ustb.edu.cn (W.L.); 2 Beijing Key Laboratory for Magneto-photoelectrical Composites and Interface Science, University of Science and Technology Beijing, Beijing 100083, China

**Keywords:** aging, DNA methylation, epigenetics, age prediction, gradient boosting regressor

## Abstract

All tissues of organisms will become old as time goes on. In recent years, epigenetic investigations have found that there is a close correlation between DNA methylation and aging. With the development of DNA methylation research, a quantitative statistical relationship between DNA methylation and different ages was established based on the change rule of methylation with age, it is then possible to predict the age of individuals. All the data in this work were retrieved from the Illumina HumanMethylation BeadChip platform (27K or 450K). We analyzed 16 sets of healthy samples and 9 sets of diseased samples. The healthy samples included a total of 1899 publicly available blood samples (0–103 years old) and the diseased samples included 2395 blood samples. Six age-related CpG sites were selected through calculating Pearson correlation coefficients between age and DNA methylation values. We built a gradient boosting regressor model for these age-related CpG sites. 70% of the data was randomly selected as training data and the other 30% as independent data in each dataset for 25 runs in total. In the training dataset, the healthy samples showed that the correlation between predicted age and DNA methylation was 0.97, and the mean absolute deviation (MAD) was 2.72 years. In the independent dataset, the MAD was 4.06 years. The proposed model was further tested using the diseased samples. The MAD was 5.44 years for the training dataset and 7.08 years for the independent dataset. Furthermore, our model worked well when it was applied to saliva samples. These results illustrated that the age prediction based on six DNA methylation markers is very effective using the gradient boosting regressor.

## 1. Introduction

Aging is an irreversible natural process in human life which is influenced by many factors, such as genetic factors, living environment and diseases [1,2]. Aging can be modified and regulated by various mechanisms at a molecular level, such as oxidative damage of DNA, chemical modification on DNA, and shortened and dysfunctional telomeres [3]. Although many methods have been used to estimate individual age, the problems of low sensitivity and prediction accuracy still to be improved [4,5,6,7]. Recent studies have shown that human aging is related to the alteration of DNA methylation in genome specific locations, and these epigenetic modifications can be used to estimate the individual age [8,9]. 

DNA methylation (DNAm) refers to the chemical modification process which transfers the active methyl to the specific base on the DNA chain under the catalysis of DNA methyltransferase (DNMT) [10]. DNA methylation can occur at the N-6 position of adenine, N-7 position of guanine, C-5 position of cytosine and so on. However, in the mammalian genome, DNA methylation often occurs on C (cytosine) of 5’-CpG-3’ to generate 5-methyldeoxycytidine (5mC). Due to the close relationship between DNA methylation and human development, tumor diseases, especially the transcriptional inactivation of tumor suppressor genes induced by CpG island methylation, DNA methylation has become an important research topic in epigenetics and epigenomics. DNA methylation is actually an epigenetic modification that plays an important modulation role in individual growth, development, gene expression patterns and the stability of the genome without changing DNA sequences [11]. In addition, this modification can be steadily transmitted in the process of development and cell proliferation [12]. Some studies have shown that the level of DNA methylation is closely related to age. With age, the DNA methylation level of the global genome is decreasing [13,14,15]. It has been reported that 5mC is increased with age in some specific CpG sites, whereas at other CpG sites, the level of 5mC decreases with age [16,17]. For some CpG sites, the degree of DNA methylation is closely related to aging, therefore it can be used for age prediction [8,18,19,20,21,22]. 

In the past, an individual’s age could be predicted by measuring and analyzing skeletal markers such as bones and teeth [23,24]. This method is limited to the existence of the skeleton. In molecular biology, DNA damage, mitochondrial mutations, and the length of leukocyte telomere are related to aging, and can also be used to predict age [25,26]. However, these methods are less accurate or are technically difficult. Furthermore, in most crime scenes, the perpetrators have fled after the crime, with only piecemeal remains such as blood, saliva or semen to be found. Therefore, it is imperative to find other feasible methods for the prediction of individual age. It has long been known that the aging process can cause changes in the molecular level of tissues and organs. It has not been found until recently that changes in DNA methylation can be used to predict age. Some reports have translated age-related DNA methylation into an age prediction model to reveal individual age [8,18,20,27,28,29]. For example, Yi et al. reported a multiple linear regression to predict age in blood samples in 2014 [30]. The model showed that the average difference between predicted age and actual age was around 4 years. Zbiec-Piekarska et al. analyzed the CpG sites in blood and built a multiple linear regression model in 2015 [31]. Based on a combination of five DNA methylation markers, the mean absolute deviation (MAD) of prediction age was 3.9 years. Huang et al. selected five age-related CpG sites from 38 candidate markers by pyrosequencing and established a linear regression model to predict age in 2015 [32]. The accuracy of their model was slightly lower, and the MAD was 7.986 years. Park et al. selected three CpG sites and used DNA methylation markers in blood from the Asian population to predict age in 2016 [33]. They identified a root mean square error (RMSE) of 6.320 years and an MAD of 3.156 years. In addition, Hannum et al. established a quantitative model with 71 highly age-related markers in 2013 [19]. The correlation coefficient between the true age and the predicted age was 0.96, and the average error was 3.9 years. However, most of these studies were based on biological experiments to identify sites. They are time-consuming and complicated to operate. Therefore, it is necessary to develop a computational method to select the candidate CpG sites. Existing models primarily use linear regression models to interpret the complex relationship between DNA methylation and age [8,30,32]. For a limited number of CpG sites, it is necessary to find a reliable age prediction model to improve the accuracy. In this study, we adopted a gradient boosting regressor to predict age, and its results were better than the existing methods. 

## 2. Materials and Methods

### 2.1. Data Collection and Processing

In this study, we obtained dozens of blood datasets from the National Center for Biotechnology Information (NCBI) Gene Expression Omnibus (GEO) (https://www.ncbi.nlm.nih.gov/geo/query/acc.cgi). All of these DNA methylation data were retrieved from two platforms, HumanMethylation27 BeadChip and HumanMethylation450 BeadChip. Some of the GEO datasets contained ethnicity information: GSE36064 (Caucasian, Chinese, and African American), GSE40279 (Caucasian, European), GSE65638 (Chinese), GSE51032 (Italycohort), GSE41169 (Dutch population), GSE27317 (African-American, Caucasian and other), GSE34257 (Gambian), GSE37008 (European, Caucasian or other ethnicity), GSE41037 (Dutch population). The datasets that did not provide the age of individuals were excluded. Finally, 25 complete datasets were obtained, of which 16 were healthy and 9 were disease datasets. The diseases which affect the DNA methylation will lead to bias in age prediction. So we divided the datasets into two categories. One was the healthy datasets (Table 1) and the other was the disease datasets (Table 2). To illustrate the performance of our model, we randomly divided each dataset into training and independent in a ratio of 7:3. The training dataset for each divided data is combined into one piece, and so is the independent dataset. A total of 1899 healthy individuals from different race backgrounds with ages between 0 and 103 years were divided into 1322 training samples and 577 independent samples. The 9 disease datasets were divided into 1673 training samples and 722 independent samples. 

### 2.2. Methylation Quality Control

To explain the common experimental biases and perform quality control analysis on DNA methylation datasets, we used principal component analysis (PCA) to identify and remove abnormal samples. To do this we used MATLAB R2014b software (v8.4.0.150421 win64) for processing. First of all, we standardized each dataset, then performed principal component analysis and extracted the first two principal components, and finally made a cluster diagram. Samples outside the circle with a radius of five were defined at outliers and removed, this filtering procedure was iteratively executed until no samples were determined to be outliers. A total of 22 healthy samples were removed and 23 disease samples were removed. 

### 2.3. Selection of Age-Related CpG Sites

For each CpG site, the β value indicates the percentage of methylation. The β value of the site is equal to one if it is fully methylated, and zero if it is completely unmethylated. There are batch effects between different data platforms. This batch effect can be partially overcome by Z-score conversion, so we used Z-score to normalize the methylation levels between different datasets to avoid obvious batch effects and used the normalized methylation values for age prediction analysis (This used the IBM SPSS v.22 software processing.) Therefore, all the DNA methylation values used the normalized β values. To identify age-related DNA methylation markers, we calculated Pearson correlations between age and DNA methylation value of each CpG site for every dataset from 1 to 103 years old (because Pearson correlation cannot be calculated for the datasets where objects have the same age). According to the Pearson correlation analysis, we chose the highly age-related r values (including positive and negative correlations) in each dataset and calculated the overlapping sites selected in each dataset. Finally, seven sites with high repetition frequency were selected. These sites were cg22736354, cg06493994, cg02228185, cg09809672, cg19761273, cg01820374 and cg19283806. Some datasets did not contain cg19283806, so it was rejected. To select the appropriate number of these sites for age prediction, we used stepwise forward to select variables and got the sequential results about the importance of markers (cg09809672, cg02228185, cg01820374, cg22736354, cg06493994, cg19761273). For this type of analysis, the markers were added to the age prediction model one by one [3]. It has been shown that the combination of these six markers had the highest accuracy. Finally, six age-related hypomethylated or hypermethylated CpG sites were determined (Table 3). Among them, cg22736354 and cg06493994 were positively correlated with age. However, cg02228185, cg09809672, cg19761273 and cg01820374 were negatively correlated with age. This is consistent with the results of Horvath’s research report [20]. To analyze the robustness of the six CpG sites, we split the data for 450K and 27K, and obtained the same sites in the 27K data. Similar results were not obtained at 450K, which may be due to 450K have relatively less data (only 5 datasets), but the selected six CpG sites had good prediction ability in subsequent prediction.

### 2.4. Algorithm

In recent years, age prediction models in blood based on a small number of CpG sites have been studied [9,27,34]. Other tissues, such as saliva [18,35], semen [36] and teeth [37] have been investigated, too. Most of these models are linear regression models. However, it is impossible to clarify the complex relationship between DNA methylation and age using a simple linear model. To minimize the prediction error and improve the accuracy of the model, the gradient boosting regressor (GBR) model has been utilized [38]. GBR is an integrated model with higher performance and better stability. Friedman proposed the GBR algorithm that extends the boosting algorithm in order to solve the regression problem. The algorithm uses the negative gradients of the loss function to solve the minimum value. GBR has been widely used in biological research, which can handle unclean and noisy data well, support different loss function, and has strong predictive ability for nonlinear data [38]. The gradient boosting regressor algorithm was executed with the sklearn package (October 2017. scikit-learn 0.19.1). It avoids the overfitting problem in decision tree learning by stopping tree growth as early as possible. The parameters of GBR are loss = ‘lad’, learning_rate = 0.03, n_estimators = 300, subsample = 0.6, λ = 0.6, min_samples_spli = 2, max_depth = 4, verbose = 1, warm_start = True. The parameters of Support Vector Regression (SVR) are kernel = ‘rbf’, degree = 3, coef = 0.0, tol = 0.001, C = 1.0, ε = 0.1. The parameters of BayesianRidge are n_iter = 300, tol = 0.001, $\alpha_{1}=10^{-6},{\;\alpha }_{2}=10^{-6},{\;\lambda }_{1}=10^{-6},{\;\lambda }_{2}=10^{-6}$.

### 2.5. Statistical Measurements

In the age prediction model, we used 1899 samples from different races and evaluated the age prediction model by calculating the MAD. The MAD is the mean absolute deviation between the predicted age and the actual age. The degree of correlation between predicted age and true age is measured by calculating $R^{2}$. All statistical analyses were done using Python 3.6 programming. They are defined as below:

$$\left\{ \begin{array}{l} MAD=\frac{\sum_{i=1}^{m}\left|y^{i}-\bar{y}\right|\;\;}{m}\\ MSE=\frac{\sum_{i=1}^{m}{\left(y^{i}-\bar{y}\right)}^{2}}{m}\\ RMSE=\sqrt{\frac{\sum_{i=1}^{m}{\left(y^{i}-\bar{y}\right)}^{2}}{m}}\\ \;R^{2}=1-\frac{\;\sum_{i=1}^{m}{\left(y^{i}-f\left(x^{i}\right)\right)}^{2}}{\sum_{i=1}^{m}{\left(y^{i}-\bar{y}\right)}^{2}} \end{array}\right.$$

where m denotes the number of target values $y={\left(y^{1},y^{2},\ldots ,y^{m}\right)}^{T},$ $\bar{y}$ is the prediction value, and $f\left(x^{i}\right)$ represents the regression function for feature vector $x^{i}.$ The MAD denotes mean absolute deviation, MSE (mean square error), and RMSE (root mean square error). 

## 3. Results

### 3.1. Healthy Blood Data Results

**Figure 1:**
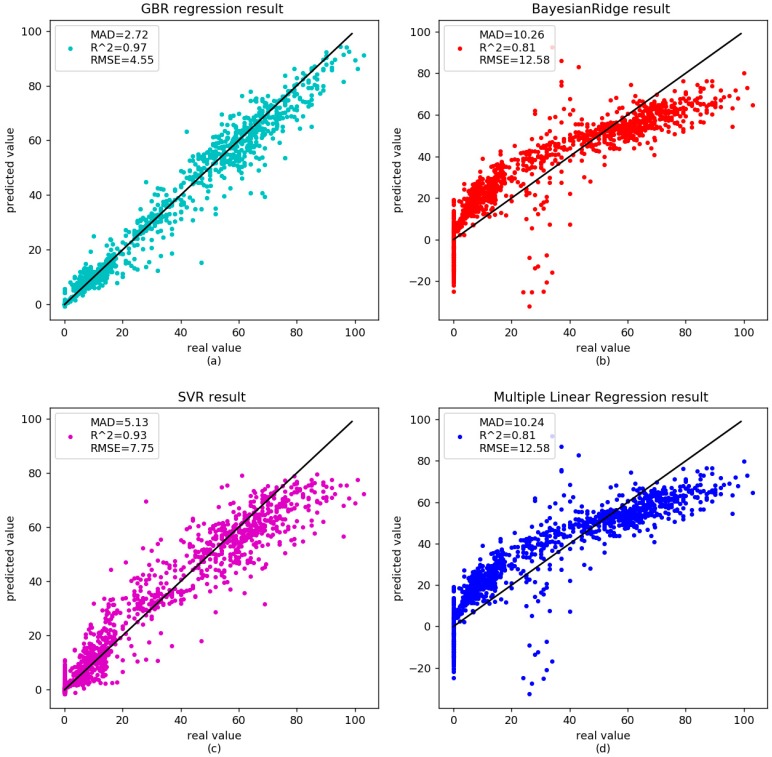
Comparison between the real age and the age predicted by the four models in the training dataset of health data. GBR: gradient boosting regresion; MAD: mean absolute deviation; RMSE: root mean square error; SVR: support vector regression.

**Figure 2:**
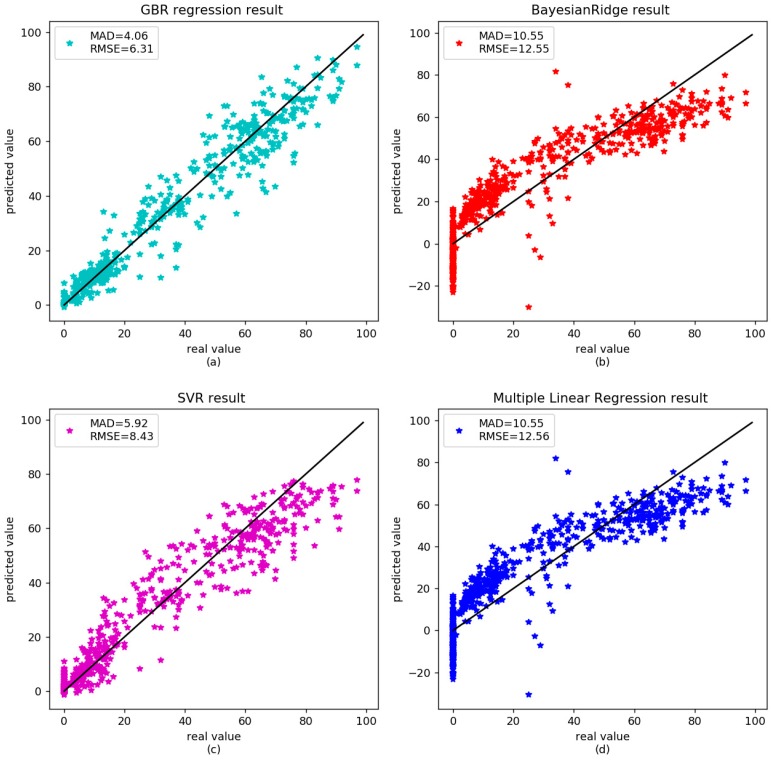
Comparison between the real age and the age predicted by the four models in the validation dataset of healthy data.

To verify the accuracy of the GBR model, three other models—BayesianRidge, Multiple Linear Regression (MLR) and SVR—were also executed. The results showed that the correlation between age and DNA methylation was 0.97 for the gradient boosting regressor, with RMSE and MAD being 4.55 and 2.72 years, respectively (Figure 1a). The RMSE and MAD were 12.58 and 10.26 years for BayesianRidge (Figure 1b), 7.75 and 5.13 years for Support Vector Regression (Figure 1c), 12.58 and 10.24 years for multiple linear regression (Figure 1d). For the independent datasets of 583 samples, the MAD was 4.06 years for gradient boosting regressor (Figure 2a), 10.56 years for BayesianRidge (Figure 2b), 5.93 years for Support Vector Regression (Figure 2c), and 10.55 years for multiple linear regression (Figure 2d). The detailed results are shown in Table 4. All the values were identified on the same CpG sites. The results showed that the prediction accuracy of the gradient boosting regressor was better than those of other linear regression models. 

### 3.2. Disease Blood Data Results

**Figure 3:**
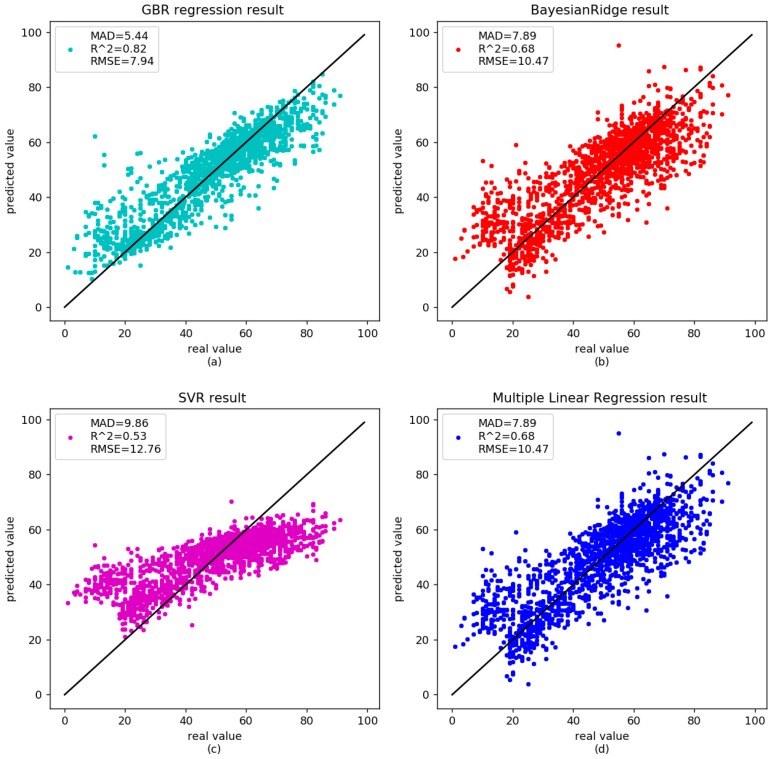
Comparison between the real age and the age predicted by the four models in the training dataset of disease data.

**Figure 4:**
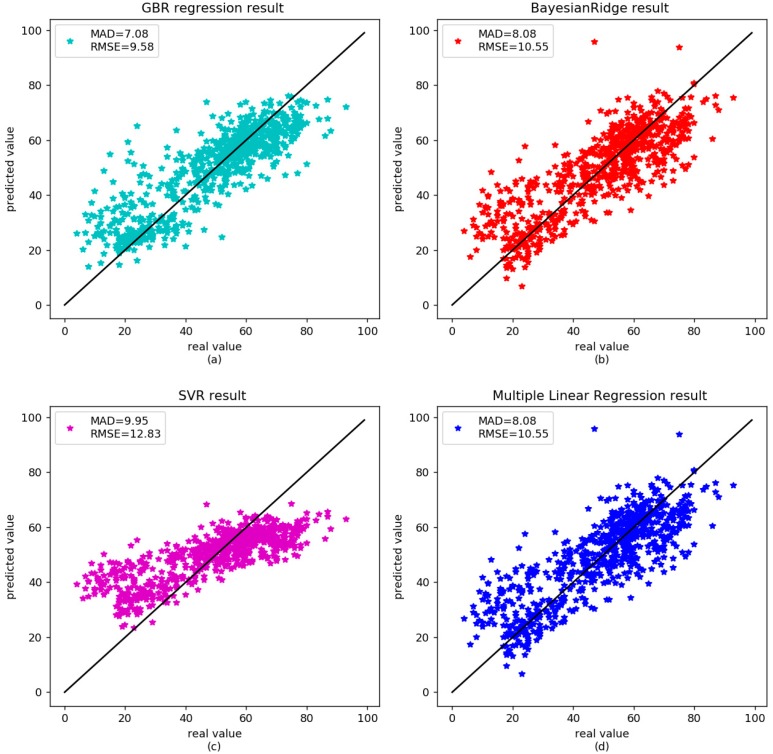
Comparison between the real age and the age predicted by the four models in the validation dataset of disease data.

There was no significant correlation between age-related methylation and sex or race [3]; however, some genes were associated with age-related diseases, such as cancer, Alzheimer’s, and so on. DNA methylation will be disordered in these diseases. Horvath et al. reported that the predicted age in cancer was poorly correlated with patient ages [20]. Park et al. found the correlation between age and methylation of three CpG sites in patients with acute myeloid leukemia (AML) disappeared [33]. Alzheimer’s disease is also known as senile dementia. The degree of methylation in the promotor region of amyloid preprotein gene declined with age in the patients [39,40]. We analyzed nine diseased samples in Table 2 to further validate the proposed GBR. The correlation between age and DNA methylation was 0.83 in our GBR. The RMSE and MAD were 7.81 and 5.91 years, respectively (Figure 3a). For the independent set, the MAD was 6.99 years (Figure 4a). The results of other models are shown in Table 5. As shown in the Table 5, the diseases affect the age prediction based on DNA methylation. However, GBR still performed well in these disease samples.

We predicted the age per disease group to see whether there would be a systematic difference between predicted age and chronological age. For this purpose, we analyzed each diseased sample. The obtained MAD for each disease was as follows: ovarian cancer was 5.91 years; type 1 diabetes mellitus (DM) was 5.33 years; Crohn’s disease or ulcerative colitis was 5.15 years; head and neck squamous cell carcinoma (HNSCC) was 7.04 years; schizophrenia was 4.54 years; rheumatoid arthritis was 4.45 years; breast cancer, colorectal cancer and other primary cancers was 6.51 years; and neurodegenerative tauopathy was 3.95 years. Neurodegenerative tauopathy and schizophrenia showed the lowest age prediction error, while HNSCC demonstrated the lowest correlation with age. All these suggest that age-related DNA methylation is accelerated in these diseases, so there would not be a systematic difference between predicted age and true age.

### 3.3. Application of the Technique to Saliva

Some studies have shown that the pattern of DNA methylation is tissue-specific [41]. Koch et al. pointed out that it was difficult to define common markers that displayed general accuracy of prediction in a variety of tissues [42]. However, methylation of certain CpG sites is not always associated with tissue specificity [43]. To test the robustness of our selected age-related CpG sites when applied to the body fluids other than the blood, we studied the methylation data of 278 saliva samples (see the Supplementary S1). The methylation values of the selected 6 CpG sites were collected from a total of 278 individuals with aged between 21 to 55 years, and 196 samples were used to train the GBR model and 82 samples were used in the independent group. The results showed that the correlation coefficient between predicted age and real age was 0.85, and the MAD was 2.1 years (training) and 5.3 years (independent). The other model results are shown in the Table 6.

To assess the performance of the GBR model, we also compared it to other studies. Bocklandt et al. identified 88 CpG sites in 80 genes [18]. Using a multiple linear regression model, the correlation coefficient between age and DNA methylation was 0.73, and the average error was 5.2 years. Using the same data (GSE28746), which included 84 individuals, the selected six sites in this work were used. The correlation coefficient between age and DNA methylation is 0.58, and the average error is 3.76 years, which is more accurate than Bocklandt’s multiple linear regression (Table 7). These results highlight the robustness of GBR model on non-blood tissue.

### 3.4. Analysis of the Selected Six CpG Sites

**Figure 5:**
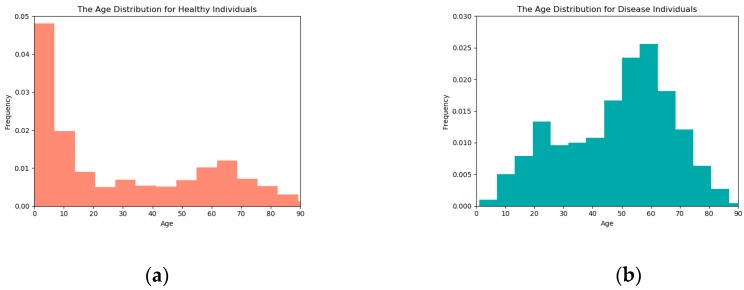
(**a**) A histogram of the age distribution for healthy individuals; (**b**) A histogram of the age distribution for disease individuals.

**Figure 6:**
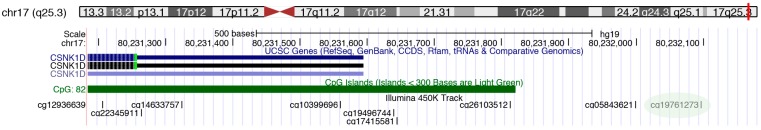
UCSC genome browser view of the genomic location of the CpG cg19761273.

In the existing studies, the ranking of age-related CpG sites is quite different. This is probably due to the difference in age range, methods and statistical techniques (the age range is shown in Figure 5). Furthermore, there is almost no overlap in calculating DNAm-based age prediction factors for different tissues. The six CpG loci extracted from the blood data can be applied to predict saliva data without any adjustment, and the prediction results are better than other predictive factors. Therefore, it is a complex task to select the CpG sites to establish the prediction age model. In this work, we selected six age-related CpG sites (AR-CpGs). These six sites are from six specific genes, including *edaradd*, *nhlrc1*, *aspa*, *lag3*, *scgn* and *csnk1d*, respectively. These special genes play important roles in regulation of developmental processes. We annotated these CpGs to their associated genes. The detailed locations of these CpGs were also included in Table 3. Two CpGs were located at the promoter region of genes (e.g., TSS1500), three were located at the first exon region and one in gene body. Meanwhile, two CpGs were located within CpG island regions, three were located at the CpG island shores, and one was far from the CpG island regulatory regions. For example, the CpG cg19761273 is located at the TSS1500 regions of the gene *edaradd* and overlapping with south shore of the CpG island, see Figure 6.

## 4. Discussion

Many bioinformatical studies have established linear regression models to study the relationship between DNA methylation and age. The reason for this is that the linear model is fast, interpretable and easy to use. However, Alisch and her colleagues et al. used non-linear models to do that in children (3–17 years old). In addition, they found that the DNA methylation did not change at a constant rate with age in life [44]. Bekaert et al. also noted that the relationship between DNA methylation and age in *elovl2* was not a straight line [37], illustrating that the linear model does not always predict age very well, and that non-linear models can sometimes be a good fit. In this study, we selected six CpG sites by calculating the Pearson correlation between age and DNA methylation values. Gradient boosting regressor was adopted, which is an integrated model. It was found that the correlation between predicted age and true age was strong ($R^{2}=0.97$). In addition, the MAD was 2.72 years. In the combined independent datasets, the MAD of age prediction was 4.06 years. The MAD value was lower than those of the other three models. This indicates that the GBR is a more suitable model for age prediction. 

Studies have shown that the level of DNA methylation is closely related to age, where most CpGs from CpG islands were highly hypermethylated during aging [13,45]. Here we observed that two of the CpG island sites were hyper-methylated, while the remaining ones showed hypo-methylation with aging, with none of them being present at CpG islands. Previous studies have shown there was no strong evidence showing DNA methylation was strongly associated with known aging-related mechanisms, but the aging-associated CpGs may represent a set of biomarkers for predicting the cellular chronological clock [3,8,46]. Specifically, we noted that majority of the genes were not presented in the previously reported genes whose expression changes with aging [46,47], but all 6 of these genes were involved in age-related processes. All CpG sites showing close correlation with age belong to genes involved in age-related processes. Here are a few examples. *edaradd* was identified by its association with ectodermal dysplasia, and specifically with hypohidrotic ectodermal dysplasia, a genetic disorder characterized by defective development of hair, teeth, and eccrine sweat glands [48]. The *nhlrc1* gene provides instructions for making a protein called malin. Although this protein is active in cells throughout the body, it appears to play a critical role in the survival of nerve cells (neurons) in the brain. The *aspa* gene provides instructions for making an enzyme called aspartoacylase. In the brain, this enzyme breaks down a compound called N-acetyl-L-aspartic acid (NAA) into aspartic acid (an amino acid which is a building block for many proteins) and another molecule called acetic acid. LAG3’s main ligand is MHC class II, to which it binds with higher affinity than CD4 [49]. The protein negatively regulates cellular proliferation, activation, and homeostasis of T cells, in a similar fashion to CTLA-4 and PD-1 [50,51] and has been reported to play a role in Treg suppressive function [52]. LAG3 also helps maintain CD8+ T cells in a tolerogenic state [53] and, working with PD-1, helps maintain CD8 exhaustion during chronic viral infection [54]. LAG3 is known to be involved in the maturation and activation of dendritic cells [55]. SCGN is a secreted calcium-binding protein which is found in the cytoplasm. It is related to calbindin D-28K and calretinin. This protein is thought to be involved in potassium chloride-stimulated calcium flux and cell proliferation [56]. The *csnk1d* gene encodes the casein kinase I isoform delta enzyme in humans [57]. This gene is a member of the casein kinase I (CKI) gene family whose members have been implicated in the control of cytoplasmic and nuclear processes, including DNA replication and repair. Interestingly, gene expressions of the selected hypo-methylated genes *aspa* and *csnk1d* were reported to be positively associated with aging [58,59], which implied potentially inverse correlations between the methylation level and the expression level to those usually occurring in promoter regions. Taken together, these genes have an important influence on the development, and their methylation could play vital roles in the regulation of aging.

Of course, our research also has some limitations. Firstly, we did not consider the impact of gender on age prediction. Some researchers have reported that age-related methylation may be different in gender [1]. However, in Bram’s study, there was no significant difference in age-related methylation level between males and females [37]. Secondly, because data on other tissues is limited, we only studied blood tissue. Each tissue has a different methylation pattern, and there is a specific methylation change during aging [60]. If more age-related methylation sites can be found in different tissues, the available methylation indicators for age prediction will be enormous. Undoubtedly, the combination of multiple age-related methylated markers will contribute to accurately estimating age. 

## 5. Conclusions

Age prediction based on DNA methylation is a rapidly evolving field of epigenetics, and it has great potential to provide accurate results. In this study, we selected six highly age-related CpG sites through calculating person correlation between age and DNA methylation value of each CpG site. By comparing the prediction effects of GBR with other linear methods, the results showed that GBR has a better prediction accuracy for blood samples. In healthy datasets, the MAD was 2.72 years for the training set and 4.06 years for the independent set. Furthermore, the age-related DNA methylation was associated with the specifically age-related diseases. The MAD clearly increased on the disease data, which was 5.44 years in the training set and 7.08 years in the independent set. GBR also achieved good results in saliva. 

## Figures and Tables

**Table 1:** Sixteen healthy DNA-methylation datasets.

DNA Origin	Platform	No.	Age Range	Author and Publication Year	Availability
Whole Blood	27K	93	(49, 74)	Rakyan (2010)	GSE20236
Blood CD4+CD14	27K	50	(16, 69)	Rakyan (2010)	GSE20242
Blood PBMC 1	27K	398	(3.6, 18)	Alisch (2012)	GSE27097
Blood Cord	27K	168	(0, 0)	Adkins (2011)	GSE27317
Blood PBMC	450K	40	(0, 103)	Heyn (2012)	GSE30870
Blood PBMC	450K	71	(3.5, 76)	Harretal (2012)	GSE32149
Blood Cord	27K	84	(0, 0)	Khulan (2012)	GSE34257
Blood Cord	27K	24	(0, 0)	Mallon (2012)	GSE34869
Blood PBMC	450K	78	(1, 16)	Alisch (2012)	GSE36064
Blood Cord	27K	123	(0, 0)	Gordon (2012)	GSE36642
Blood Cord	27K	48	(0, 0)	Turan (2012)	GSE36812
Blood PBMC	27K	91	(24, 45)	Lam (2012)	GSE37008
Whole Blood	450K	500	(26, 101)	Hannum (2012)	GSE40279
Whole Blood	450K	95	(18, 65)	Horvath (2012)	GSE41169
Whole blood	450K	43	(47, 59)	Bell (2013)	GSE53128
Blood	450K	16	(21, 32)	Xu (2015)	GSE65638

^1^ Peripheral blood mononuclear cell.

**Table 2:** Nine disease DNA-methylation datasets.

DNA Origin	Platform	No.	Age Range	Author and Publication Year	Availability
Whole Blood	27K	203	(50, 85)	Song (2010)	GSE19711
Whole Blood	27K	194	(1, 32)	Teschendorff (2010)	GSE20067
Peripheral Blood	450K	46	(3.5, 76)	Harris (2011)	GSE32148
Blood	450K	24	(52, 88)	Athanasios (2012)	GSE40005
Whole Blood	27K	498	(16, 86)	Horvath (2012)	GSE41037
Whole Blood	450K	500	(18, 70)	Liu (2013)	GSE42861
Blood	27K	71	(23, 85)	Day (2013)	GSE49904
Blood	450K	499	(34, 72)	Polidoro (2013)	GSE51032
Peripheral Blood	450K	383	(34, 93)	Lwe (2013)	GSE53740

**Table 3:** Information of 6 selected age-related CpG sites.

CpG ID	Gene ID	Chromosome Location ^1^	Gene Region ^2^	Relation to GpG Island ^3^	Correlation Status	Reference
cg09809672	EDARADD	1:236557682	TSS1500	N_Shore	Negative	[1,17,33]
cg22736354	NHLRC1	6:18122719	1stExon	Island	Positive	[2,7,18,19]
cg02228185	ASPA	17:3379567	1stExon	--	Negative	[7,26,33]
cg01820374	LAG3	12:6882083	Body	N_Shore	Negative	[1]
cg06493994	SCGN	6:25652602	1stExon	Island	Positive	[2,7,18,19]
cg19761273	CSNK1D	17:80232096	TSS1500	S_Shore	Negative	[2]

^1^ Chromosome location is referred to the Human genome reference GRCh37 version. ^2^ TSS: transcription start site. TSS1500: 1500 bp flanking region from the TSS. ^3^ CpGs island table were downloaded from University of California Santa Cruz (UCSC) browser. Distance of 2kb to CpG islands were defined as CpG island shores (N_Shore: downstream of CpG island and S_Shore: up-stream of the CpG island).

**Table 4:** Comparison of gradient booster regressor (GBR) with the other three methods on healthy datasets.

	R^2^	MAD	MSE	RMSE
Training				
Gradient Boosting Regressor	0.9747	2.7171	20.7243	4.5524
BayesianRidge	0.8055	10.2561	158.3044	12.5819
Support Vector Regression	0.9267	5.1338	60.0420	7.7487
Multiple Linear Regression	0.8055	10.2448	158.2800	12.5809
Testing				
Gradient Boosting Regressor	0.9523	4.0593	39.8269	6.3109
BayesianRidge	0.8101	10.5654	157.8721	12.5647
Support Vector Regression	0.9151	5.9267	71.2060	8.4384
Multiple Linear Regression	0.8104	10.5510	157.6726	12.5568

MAD: mean absolute deviation; MSE: mean square error; RMSE: root mean square error.

**Table 5:** Results comparison of GBR with the other three methods on disease datasets.

	$R^{2}$	MAD	MSE	RMSE
Training				
Gradient Boosting Regressor	0.8186	5.4401	63.0648	7.9413
BayesianRidge	0.6844	7.8944	109.6227	10.4701
Support Vector Regression	0.5333	9.8583	162.6949	12.7552
Multiple Linear Regression	0.6844	7.8946	109.6222	10.4701
Testing				
Gradient Boosting Regressor	0.7374	7.0832	91.7887	9.5806
BayesianRidge	0.6812	8.0786	111.2896	10.5494
Support Vector Regression	0.5303	9.9573	164.6747	12.8326
Multiple Linear Regression	0.6812	8.0795	111.3016	10.5500

**Table 6:** Results comparison of GBR with the other three methods on saliva datasets.

	R^2^	MAD	MSE	RMSE
Training				
Gradient Boosting Regressor	0.8539	2.1040	13.7795	3.7121
BayesianRidge	0.4310	5.7483	52.5169	7.2469
Support Vector Regression	0.0227	7.9369	99.5273	9.9763
Multiple Linear Regression	0.4333	5.6775	52.3045	7.2322
Testing				
Gradient Boosting Regressor	0.4298	5.3478	56.1291	7.4919
BayesianRidge	0.5423	5.5389	43.8468	6.6217
Support Vector Regression	0.0308	8.4729	104.4403	10.2196
Multiple Linear Regression	0.5479	5.4662	43.3933	6.5874

**Table 7:** Results of GBR and Multiple Linear Regression on saliva samples.

	No. of CpG Sites	$R^{2}$	MAD
Multiple Linear Regression	88	0.73	5.2
Gradient Boosting Regressor	6	0.58	3.76

## Supplementary Materials

The following are available online at http://www.mdpi.com/2073-4425/9/9/424/s1, Supplementary S1: The 278 saliva samples.
